# High pneumonia lifetime-ever incidence in Beijing children compared with locations in other countries, and implications for national PCV and Hib vaccination

**DOI:** 10.1371/journal.pone.0171438

**Published:** 2017-02-06

**Authors:** Fang Qu, Louise B. Weschler, Yuexia Sun, Jan Sundell

**Affiliations:** 1 China Meteorological Administration Training Centre, China Meteorological Administration, Beijing, China; 2 Department of Building Science, Tsinghua University, Beijing, China; 3 Independent Researcher, Colts Neck, New Jersey, United States of America; 4 School of Environmental Science and Engineering, Tianjin University, Tianjin, China; Imperial College London, UNITED KINGDOM

## Abstract

**Objectives:**

To compare the proportion of Beijing children who have ever had pneumonia (*%Pneumonia*) to those in other locations, and to estimate by how much national vaccine coverage with Pneumococcal Conjugate Vaccine (PCV) and Haemophilus Influenzae Type b (Hib) could reduce Beijing *%Pneumonia*.

**Methods:**

*%Pneumonia* was obtained for each age group from 1 to 8 years inclusive from 5,876 responses to a cross-sectional questionnaire. Literature searches were conducted for world-wide reports of *%Pneumonia*. Previous vaccine trials conducted worldwide were used to estimate the pneumococcal (*S*. *pneumoniae*) and Hib (*H*. *influenzae)* burdens and *%Pneumonia* as well as the potential for PCV and Hib vaccines to reduce Beijing children’s *%Pneumonia*.

**Findings:**

The majority of pneumonia cases occurred by the age of three. The cumulative *%Pneumonia* for 3–8 year-old Beijing children, 26.9%, was only slightly higher than the 25.4% for the discrete 3 year-old age group, similar to trends for Tianjin (China) and Texas (USA). Beijing’s *%Pneumonia* is disproportionally high relative to its Gross National Income (GNI) per capita, and markedly higher than *%Pneumonia* in the US and other high GNI per capita countries. Chinese diagnostic guidelines recommend chest X-ray confirmation while most other countries discourage it in favor of clinical diagnosis. Literature review shows that chest X-ray confirmation returns far fewer pneumonia diagnoses than clinical diagnosis. Accordingly, Beijing’s *%Pneumonia* is likely higher than indicated by raw numbers. Vaccine trials suggest that national PCV and Hib vaccination could reduce Beijing’s *%Pneumonia* from 26.9% to 19.7% and 24.9% respectively.

**Conclusion:**

National PCV and Hib vaccination programs would substantially reduce Beijing children’s pneumonia incidence.

## Introduction

By 2012, Beijing had achieved a remarkable decrease in her mortality rate for children under 5 years old (U5MR) to 3.6 per thousand (3.6‰) [1] compared to 7.1‰ for the United States [2,3]. However, the Beijing pneumonia U5MR of 0.25‰ in 2012 [1], was still higher absolutely and more especially as a fraction of the total U5MR than the 0.20‰ in 2012 for the United States [2]. Pneumonia, the world’s largest killer of children from 1 to 59 months, persists as a severe public health problem in Beijing [4].

Although pneumonia is said to be a major public health problem in China [5], pneumonia incidence data for China have been scarce [5–7]. Total pneumonia incidence cannot be deduced from hospitalization and/or mortality rates. The present study, part of the China, Children, Homes, Health (CCHH) multi-city project, used a questionnaire that asked parents of kindergarten children (1 to 8 years old) whether their child had ever had doctor-diagnosed pneumonia [8]. Thus, our study returned total incidence as well as the proportion of children in each age group from 1 to 8 who had ever had pneumonia. We will use *%Pneumonia* to designate the proportion of Beijing children who have ever had pneumonia. To our knowledge, these are the first such estimates for Beijing children. Of great interest is how Beijing children’s *%Pneumonia* compares with others around the world. We note that such comparisons require accounting for diagnostic methods because diagnosis using chest X-ray confirmation returns many fewer pneumonia diagnoses than clinical judgment [4,9].

Developed countries are widely acknowledged to have much lower pneumonia incidence than developing countries [3,4]. The World Bank defines “developed” as having Gross National Income (GNI) per capita equal to or above the “high income” threshold. In 2014, while China’s overall GNI per capita was 60% of the World Bank’s $12,736 threshold for high income [10], the provincial level municipality of Beijing had achieved near developed status with a GNI per capita calculated at 98% that of the high income threshold [10,11] (calculated below in the section “Relative GNI”) [11,12].

Viral, bacterial and other micro-organisms cause pneumonia [4]. There are vaccines for two of the bacterial pathogens, “Haemophilus Influenzae Type b” (Hib) for *H*. *influenzae* type B [4,13] and “Pneumococcal Conjugated Vaccine” (PCV) for 13 serotypes of *S*. *pneumoniae* (also called “SP” or “pneumococcus”) [4,13,14]. To avoid confusion about terminology, we note that both the *H*.*influenzae* type B pathogen and the vaccine are commonly called Hib. The Hib vaccine was first introduced in a national vaccine program by Iceland in 1989 [15], and PCV7 and PCV13 vaccines by the United States in 2000 [15] and 2010 respectively [16]. Hib is presently included in the national vaccination programs of 185 countries, and PCV7 or PCV13 in 103 countries, but as of January 2016 neither PCV or Hib was part of China’s national vaccination program [15].

Accordingly, the objectives of the present study are (1) to compare *%Pneumonia* for Beijing children to those in other regions and countries, and analyze these comparisons in the context of different diagnostic criteria, national vaccination status and GNI per capita and (2) to estimate by how much Beijing children’s *%Pneumonia* could be reduced by national coverage with PCV and Hib.

## Methods

### Acquisition of Beijing, Tianjin and Texas data

The Beijing CCHH cross-sectional survey was conducted in 2010–2011. Its methods are described in Qu et al. 2013 [17] and the full CCHH questionnaire is published in Zhang et al. 2013 [8]. The Texas and Tianjin studies used the same methods. The question for pneumonia is: *Has your child been diagnosed with pneumonia by a doctor*? *(Yes/No*). We will use *%Pneumonia* to represent the proportion of children, at a given age, who have ever had pneumonia.

### Worldwide lifetime-ever pneumonia incidence (*%Pneumonia*)

We conducted a literature search for *%Pneumonia* in other regions and countries. Inclusion criteria required (1) age-specific *%Pneumonia* and (2) total doctor-diagnosed *%Pneumonia*, regardless of whether hospitalization was required and (3) data for any group of children 3 years old and older. Nine studies, with data for 23 different locations, met these criteria (Table 1, Results and Discussion).

**Table 1:** Lifetime-ever pneumonia (*%Pneumonia*) reported for children ≥ 3 years old.

Location	Age (Years)	*N*	*%Pneumonia* (%)	Diagnostic criteria	Vaccination^a^	Reference
Changsha, China	3–6	2,622	38.3	Chest X-ray	Unknown (very low)	Lu 2014 [20]
Shanghai, China		14,084^b^ [21]	33.2		11.4% PCV, 41% Hib [18]	Zhang 2013 [8]
Chongqing, China		5,092	31.3		Unknown (very low)	
6 CCHH cities^c^, China		~19,000	31.1		Unknown (very low)	
**Tianjin, China**		4,616	29.1		5.3% PCV, 41% Hib [18]	**Present Study**
Romania	7–11	3,470	27.9	Unknown	No	Leonardi 2002 [22]
**Beijing, China**	3–6	5,331	26.9	Chest X-ray	1.2% PCV, 41% Hib [18]	**Present Study**
Bulgaria	7–11	3,631	24.7	Unknown	No	Leonardi 2002 [22]
Hungary		3,479	24.6			
Poland		2,932	21.5			
Slovak Republic		3,038	16.3			
Czech Republic		3,479	16.2			
Germany	6	2,234	13.5	Clinical	Hib	Schnabel 2009[23]
**Texas, USA**^d^	3–6	1,523	13.1		53% PCV, >90% Hib [24]	**Present Study**
Germany^e^	3	~2,100	8.8		>90% Hib	Schnabel 2009 [23]
Tucson AZ, USA	3	788	7.4	Chest X-ray	No	Castro-Rodriguez 1999 [25]
Germany^f^	7	~74,000	6.9	Clinical	>90% Hib	Weigl 2003 [26]
West Sydney, Australia	5–15	2,020	6.8	Chest X-ray	>90% Hib	MacIntyre 2003 [27]
Spain	5	654	3.0	Clinical	No	Garcés-Sánchez 2005 [28]

^a^ Shanghai, with GNI equal to 1.02 times the World Bank high income index, was reported to have 11.4% PCV and 41% Hib coverage in 2012. Given the high purchase price for these vaccines [18], we estimated that Beijing and Tianjin with relative GNI equal to 0.98 and 0.85 of the high income index had similar coverage (corrected for vaccine availability), but that Chinese cities with lower GNI had very little coverage.

^b^ Calculated from Liu 2014 [21].

^c^ Harbin, Nanjing, Taiyuan, Urumqi, Xi’an, Wuhan.

^d^ Vaccination coverage for Texas sample estimated from Center for Disease Control [24].

^e^ Munich, Leipzig, Wesel, Bad Honnef.

^f^ Schleswig-Holstein.

### Age groups

For a specific age group, *%Pneumonia* is given by:

$$\%Pneumonia=\frac{n_{\text{lifetime}}}{N}\times 100$$ (1)

where *n*_lifetime_ is the number of children in that age group who have ever had pneumonia, that is, the number of children who have had at least one episode of pneumonia, and *N* is the total number of children in that age group. Because a child can have more than one case of pneumonia, the total number of pneumonia cases for a population is greater than *%Pneumonia*.

Pneumonia incidence, defined as the number of cases in a population of children, is highest in the first 2–3 years (S1 Table). We hypothesized that *%Pneumonia*, defined as the proportion of children who have ever had pneumonia, increases from 0 to 3 years-old and then plateaus. If so, *%Pneumonia* can be compared among any samples of children between 3 and 8 years-old, with the understanding that that there are slight *%Pneumonia* increases for successive ages. For samples of children <3 years-old, *%Pneumonia* can be compared only among identical age groups. That is, for age *i* where 3≤ *i* ≤8, *%Pneumonia* can be approximated as:

$$\%Pneumonia=\frac{n_{3}+\ldots +n_{\text{i}}}{N_{3}+\ldots +N_{\text{i}}}\times 100$$ (2)

We test this hypothesis in the Results and Discussion section (*%Pneumonia* related to age).

### Diagnostic criteria

Pneumonia is diagnosed predominantly by chest X-ray confirmation of clinically suspected pneumonia, or by clinical signs and symptoms alone. We used the country’s national diagnostic guidelines as default if a study did not report diagnostic criteria.

### Vaccination status

National vaccination status for PCV and Hib was obtained from the International Vaccine Information Center [15]. We assumed full coverage if a national vaccination program had been established at least one year before the birth year of the oldest children. In China, PCV and Hib became available for private purchase in October 2008 and 2000 respectively [18]. We estimated Beijing children’s private vaccination rates using data compiled by Wagner et al. for Shanghai children [18]. We reasoned that the two cities’ similar GNIs per capita, estimated using data from the World Bank [10,11], the China National Bureau of Statistics [12] and the International Money Fund (IMF) [12] means that similar proportions of parents would be willing and able to incur the high cost of these vaccinations. Of Shanghai children born between 2005 and 2010, 11.4% received PCV7 and 41% received Hib vaccination. For Beijing children in the present study, only 10.4% of the 3 to 8 year-old Beijing children were born late enough to have had timely PCV vaccination, so PCV coverage is estimated at 1.2% (that is, 10.4% × 11.4%), leaving 98.8% of Beijing children not PCV vaccinated. All of the Beijing children were born after 2000 when Hib was available, so we estimate 41% were Hib vaccinated and 59% were not. For Tianjin children, 47% were born when PCV7 was available, so we estimate that 5.3% coverage (that is, 47% × 11.4%). All Tianjin children were born when Hib was established, so we estimate 41% Hib coverage.

### Relative GNI

Every year, the World Bank sets GNI per capita index numbers for low, lower middle, upper middle and high income [10]. A country whose GNI is equal to or more than the high income index is classified as “developed.” Accordingly, we calculated a relative GNI as the ratio of a country’s average GNI per capita for the children’s birth years to the average high income index for those years [10], so that a country’s whose relative GNI per capita ≥1.0 can be considered as “developed” [10]. GNIs were not directly available for the province-level municipalities Beijing, Tianjin, Chongqing and Shanghai, so we estimated these GNIs from their GDPs [12] multiplied by China’s GNI/GDP ratio [10,12]:

$$GNI_{\text{m}}=\left(\frac{GNI_{\text{C}}}{\;GDP_{\text{C}}}\right)\left(GDP_{\text{m}}\right)$$ (3)

where the subscript “m” denotes municipalities and the subscript “C” denotes China. These estimates assume that the municipality’s GNI/GDP ratio scales linearly with that of China.

## Results and discussion

The Beijing cross-sectional survey yielded 5,876 completed questionnaires, a 65% response rate. The 5,440 Beijing children aged 3 to 8 had an average *%Pneumonia* of 26.9%, with diagnoses most likely confirmed by chest X-ray, as per China’s national guidelines [7,19]. Table 1 shows that the *%Pneumonia* for the Chinese cities in recent years is the highest, followed by those of Central European countries (Bulgaria, Czechoslovakia, Hungary, Poland, Romania and Slovakia), whose *%Pneumonia* in the 1980s were only slightly lower than for China cities. The lowest *%Pneumonias* are for locations in the US, Germany, Australia and Spain in various years.

### *%Pneumonia* related to age

**Fig 1:**
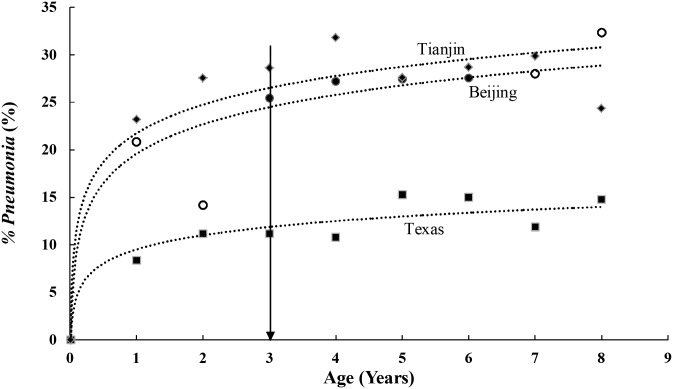
Lifetime-ever pneumonia (*%Pneumonia*) for each discrete age group from 1 to 8 years old in Beijing, Tianjin (China) and Texas (USA). Data for this figure is shown in S2 Table. The open circles for Beijing indicate small sample sizes.

Is it valid to compare *%Pneumonia* for any age group ≥3 as we have done in Table 1? We plotted *%Pneumonia* for age groups 1–8 years from Beijing, Tianjin and Texas in Fig 1, the data for which are shown in S2 Table. Fig 1 shows that *%Pneumonia* climbs steeply in the first two to three years, plateauing at about 36 months (3 years), or approximately 25% for Beijing, 10% for Texas, and 28% for Tianjin. Published data from the CCHH cities Xi’an [29] and Changsha [20] also show *%Pneumonia* increasing until age three, and then plateauing. These data are, to the best of our knowledge, the total of available *%Pneumonia* data for each age group.

For incidence, abundant data are available (S1 Table), and an example of incidence versus age for half-year age groups is shown in S1 Fig. In this scatterplot, the incidence of Lower Respiratory Tract Infection rises steeply until 3 years old and then plateaus, similarly to that of *%Pneumonia*. We conclude that *%Pneumonia* can be compared for any two years between 3 and 8 inclusive, recognizing that each subsequent year (or added year) will increase *%Pneumonia* slightly. *%Pneumonia* comparisons between groups younger than 3 years-old can only be made between same-age groups.

### No diagnostic “gold standard”

Table 1 specifies whether *%Pneumonia* was obtained using clinical judgment of signs and symptoms or chest X-ray confirmation of clinical suspicion. The national guidelines for pneumonia diagnosis in China require chest X-ray confirmation and at least one of four clinical signs or laboratory findings [5,19]. By contrast, most countries represented in Table 1, and in particular the UK [30] and US [31], recommend pneumonia diagnosis solely by clinical criteria, except in the case of severe pneumonia, where serial chest X-rays are recommended to track improvement.

There is no diagnostic “gold standard” for pneumonia [4,32,33]. We surveyed literature studies in which each subject was diagnosed separately by clinical signs/symptoms and chest X-ray (Table 2). The ratios for clinical to chest X-ray diagnoses range from 1.1 to 7.7, clustering between 2 and 3. In other words, just 1/3 to 1/2 those diagnosed with pneumonia using clinical signs had their pneumonia confirmed by chest X-ray. Accordingly, differences between *%Pneumonia* in Chinese cities (diagnosis by X-ray) and locations where diagnosis was by clinical signs are even greater than those indicated by the raw numbers of Table 1. S1 Text (Pneumonia diagnosis) further explores the difficulties associated with diagnosing pneumonia.

**Table 2:** Ratio of clinical to chest X-ray diagnoses in various samples of children.

*N*	Age	Location	Ratio of diagnoses: clinical/chest X-Ray	Comments	Reference
222	0-60M^a^	Sevagram Wardha, central India	1.05	Chest X-ray criteria were more sensitive than the WHO-EPC introduced in 2001.	Gupta 1996 [34]
2,071	<21Y^b^	Boston MA, USA	1.2	1,501 < 5 years old. Ratio is for physician rated probability of pneumonia >75%. Study conducted at Children's Hospital of Boston.	Neuman 2010 [35]
155	≤19Y	Baltimore MD and Columbus OH, USA	1.4	62% < 2 years old.	Grossman 1988 [36]
314	<60M	Hong Kong	1.8	Hospitalized. Clinical diagnosis: bacterial, pneumonia; chest X-ray criterion: consolidation.	Chiu 2014 [37]
191	<60M	Orlando FL, USA	1.9		Rothrock 2001 [38]
420	2-59M	Gambia (rural)	1.9		Kuti 2014 [39]
651	<24M	Mozambique (rural)	2.3		Roca 2010 [40]
4,093	1-35M	Bogotá, Colombia	2.4		Benavides 2012 [41]
125,983^c^	<60M	Washington, USA	2.4		Nelson 2008 [9]
100	<16Y	Unugu, Nigeria	2.7	Clinical diagnostic criteria were not specified.	Njeze 2011 [42]
13,026	1-35M	Goiana, Brazil	2.8	Goiana, Brazil, children who presented at hospital, but were not necessarily hospitalized.	Andrade 2012 [43]
1,068	<36M	Northern California	2.8	“High suspicion” of pneumonia from clinical exam. (See Black [44] below for “low suspicion” pneumonia.)	Black 2002 [44]
570	12M-16Y	Ontario or Quebec, Canada	2.8	Study includes all who presented to hospital; 97% were not admitted.	Lynch 2004 [45]
30,397^d^30,444^e^	0-17M	Guatemala (rural)	2.8	Parents excluded some clinically diagnosed children from X-ray, so ratio may be artifactually large.	Smith 2011 [46]
525	1M-16Y	Tel Aviv, Israel	2.9	68% <6 years old.	Ayalon 2013 [47]
351	≤18M	Guatemala (rural)	3.0	Children with WHO criteria for pneumonia referred to MD by field health worker for diagnosis and X-ray read by same MD.	Bruce 2007 [48]
NR^f^	<24M	Refugee camp, Thailand	3.3	Cohort was 955 children. Incidence reported as cases/(child·year).	Turner 2013 [49]
1,608	1-36M	San Jose, Costa Rica	3.5	Prospective Surveillance of children presenting to health centers for invasive pneumococcal disease only.	Arguedas 2012 [50]
413	36-60M	Hong Kong	4.1		Ho 2007 [51]
1,698	<36M	Northern California	4.5	“Lower suspicion” pneumonia (See Black [44] above for “high suspicion” of pneumonia).	Black 2002 [44]
477	<24M	Hong Kong	4.9		Ho 2007 [51]
711	0-15Y	Northern England	5.0	80% < 5years old.	Clark 2007 [52]
1,519	<60M	Pakistan (urban)	5.8	Clinical impression was "suspicion" of pneumonia.	Hazir 2006 [53]
1,622	<60M	Boston MA, USA	5.9	Clinical diagnosis based on WHO tachnypea.	Shah 2010 [54]
200	<60M	Beer Shiva, Israel	6.1	Clinical impression was “suspicion” of pneumonia.	Ben Shimol 2012 [55]
1,918	4-23M	Ukraine (urban)	7.7		Pilishvili 2013 [56]

^a^ Months.

^b^ Years.

^c^ Child·years of observation.

^d^ Child weeks of observation, clinical diagnoses.

^e^ Child weeks of observation, chest X-ray diagnoses.

^f^ Not reported, but 488/955 (51.1%) had at least one episode.

### *%Pneumonia* using chest X-ray: Beijing, Tianjin and Tucson (USA)

Table 3 shows *%Pneumonia* generated by chest X-ray (CXR) diagnoses for Beijing, Tianjin, and Tucson AZ, USA [25]. We compared 3 year-olds only so as to minimize uncertainty inherent in comparing different age groups. The *%Pneumonias* for the Chinese cities are substantially higher than that for the USA city. Table 3 does not include the 11.2*%Pneumonia* from a study in northeast Texas USA of 384 three-year-olds born in 2004 [57], because diagnosis was primarily by clinical criteria. Since *%Pneumonia* by clinical diagnosis is reliably higher than by chest X-ray confirmation, the Texas *%Pneumonia* is consistent with the Tucson data.

**Table 3:** Comparison of chest X-ray (CXR) generated *%Pneumonia* for 3 year-old children in two Chinese cities and one US location.

Location	Children’s birth years	*N*	Vaccination^a^		*%Pneumonia* (%) (Chest X-ray)	Reference
			Hib (%)	PCV (%)		
**Beijing, China**	2007–2008	1,336	41^b^	1.2^b^	**25.4**	**Present Study**
**Tianjin, China**	2009	528	41^b^	5.3^b^	**28.6**	**Present Study**
Tucson AZ, USA	1980–1984	888	No^c^	No^c^	**7.4**	Castro-Rodriguez 1999 [25]

^a^ Vaccine coverage is assumed if a national program was initiated one or more years before children’s birth.

^b^ Vaccination rates estimated from Shanghai voluntary vaccination rates [18], as explained in Methods.

^c^ Study completed before vaccines were available.

### Estimating by how much national PCV and Hib coverage can reduce Beijing children’s pneumonia

Pathogens other than *S*. *Pneumoniae* and *H*. *Influenzae*, for example viruses, can cause pneumonia—especially Respiratory Syncytial Virus (RSV) but also influenza virus, various bacteria and other organisms [4]. The etiological causative agent is usually very difficult to identify [4] so that the “etiological fraction” (the proportion of pneumonia attributable to any given pathogen [58]) cannot be determined directly. Nonetheless, vaccine trials, both Randomized Controlled Trials (RCTs) and observational studies of vaccination, measure the proportional reduction of pneumonia [13,58–61]. Such a trial, termed “Vaccine Probe” [13,58–62] estimates Vaccine Efficacy (*VE*) as well as the lower bound of etiological fraction. For any given Vaccine A that targets Pathogen A,

$$VE_{\text{A}}=\frac{I_{\text{u}}-I_{\text{v},\text{A}}}{I_{\text{u}}}\;\leq Pathogen\;A\;etiological\;fraction$$ (4)

where *I*_u_ is the incidence in the unvaccinated “control” sample and *I*_v_ is the incidence in the vaccinated “test” sample [58,59]. Re-arranging yields the incidence in a vaccinated population:

$$I_{\text{v},\text{A}}=I_{\text{u}}\left(1-VE_{\text{A}}\right)$$ (5)

While Vaccine Probe studies of PCV and Hib have not yet been conducted in China, they have been conducted in countries that differ from each other demographically and climatically, and have yielded remarkably tight ranges for both *S*. *Pneumoniae* and *H*. *Influenzae* etiological fractions, with chest X-ray confirmed pneumonia as the endpoint. Table 4 shows *VE*s calculated via meta-analysis of PCV and Hib studies. Information on the individual studies used for these meta-analyses is given in S3 Table (S3A and S3B Table). These *VE*s are sufficiently robust to estimate by how much PCV and Hib vaccination could reduce Beijing children’s *%Pneumonia*.

**Table 4:** *VE*s obtained by meta-analyses for PCV and Hib.

Study	Vaccine	Number of studies included	Countries	*VE* (%) (95% Confidence Interval, CI)
Lucero 2009 [63]	PCV	6	South Africa (2), USA (2), Finland (1), Philippines (1)	27 (15, 36)
O’Brien 2009 [14]	PCV^a^	4	Gambia, USA, Philippines, South Africa	36 (16, 52)^b^
Theodoratou 2009 [13]	PCV9	3	Gambia, South Africa, Philippines	26 (12, 37)
Theodoratou 2009 [13]	Hib	6	Bangladesh, Brazil, Chile, Colombia, Gambia, Indonesia	18 (-2, 33)

^a^ Adjusted for PCV Valence.

^b^ Confidence Interval (CI) estimated from O’Brien 2009, Fig 1.

In Table 5, we have estimated *I*_v_ for PCV (*I*_v,PCV_) and Hib (*I*_v,Hib_) using Eq (5) and the Lucero value for *VE*_*PCV*_, 27% (15%, 36%) [63] and the Theodoratou value for *VE*_Hib_, 18% (-2%, 33%) [13]. *I*_u_ = *%Pneumonia* = 26.9%. For a population in which there has been no vaccination, *%Pneumonia* would decrease from 26.9% to 19.6% for PCV or to 22.1% for Hib. Among Beijing’s 122,747 children born in 2012, 8,915 (PCV) or 5,943 (Hib) fewer children would get pneumonia. Based on Shanghai data [18], we estimate 1.2% and 41% of Beijing’s children have been vaccinated with PCV and Hib respectively. Since these children are already protected, we subtract these subsets from the total and so obtain a smaller reduction in *%Pneumonia*, from 26.9% to 19.7% for PCV or to 24.9% for Hib. Among the Beijing 2012 birth cohort, 8,808 (PCV) or 3,507 (Hib) fewer children would get pneumonia.

**Table 5:** Impact of PCV and Hib vaccination on a birth cohort of 122,747, the number of babies born in Beijing in 2012 [1], assuming *%Pneumonia* to be 26.9% at age 3. We used the Lucero value for *VE*_PCV_, 27% (15%, 36%) [63] and the Theodoratou value for *VE*_Hib_, 18% (-2%, 33%) [13]. Potential pneumonia reductions are given for both wholly unvaccinated and vaccinated populations as we have estimated for Beijing.

Vaccine	% Vaccinated	*N*_T,u_	Vaccination reduces to *%Pneumonia* from 26.9% this %.^a^ (95% CI)	This many fewer children get pneumonia (95% CI)
PCV	0	122,747	19.6 (22.9, 17.2)	8915 (4953, 11887)
Hib	0	122,747	22.1 (26.9, 21.7)	5943 (0, 10896)
PCV+Hib	0	122,747	14.8 (22.9, 12.1)	14,858 (4953, 22783)
PCV^b^	1.2	121,274	19.7 (22.9, 17.3)	8808 (4893, 11744)
Hib^b^	41	72,421	24.9 (26.9, 21.7)	3507 (0, 6429)
PCV+Hib	-	-	16.9 (22.9, 12.1)	12,315 (4893,18173)

^a^*I*_v,PCV_ = *I*_u_ (1 − *VE*_PCV_). *I*_v,Hib_ = *I*_u_ (1 − *VE*_Hib_). I_v,PCV + Hib_ = (*n*_T,u_ − *n*_T,v_)/*N*_T_.*VE*_PCV_: 27% (95%CI: 15%, 36%) [63]. *VE*_Hib_: 18% (95%CI: -2%, 33%) [13].

^b^ Estimated using data from Shanghai [18], and assuming that Beijing voluntary vaccination rates are approximately the same as those in Shanghai.

Of great interest is the impact of introducing both PCV and Hib. There are no studies to guide a prediction of this impact. However, we can propose a Confidence Interval (CI): the lower limit of *%Pneumonia* reduction is equal to or slightly greater than that of PCV alone and the upper limit is the sum of PCV and Hib reductions. Table 5 shows that for a population with no vaccination, the maximum reduction by PCV + Hib in *%Pneumonia* would be to 14.8% from 26.9%. For Beijing’s 122,747 children born in 2012, 14,900 fewer children would get pneumonia. For a population that had 1.2% PCV and 41% Hib vaccination rates, the maximum reduction in *%Pneumonia* would be to 16.9% from 26.9%. For Beijing’s 122,747 children born in 2012, 12,300 fewer children would get pneumonia.

### *%Pneumonia* related to GNI per capita

**Fig 2:**
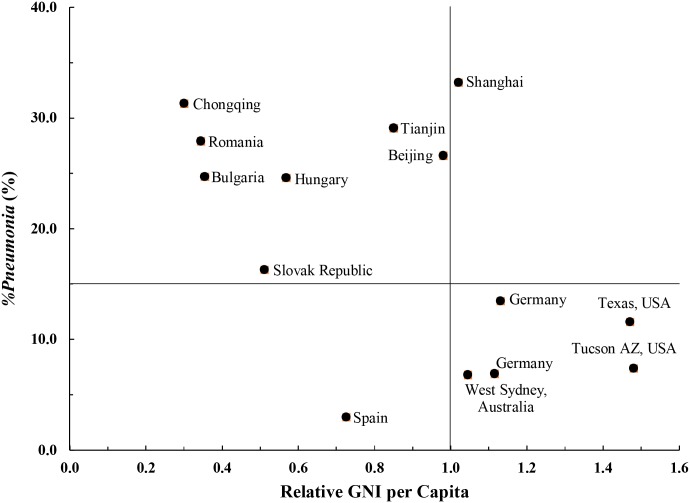
Lifetime-ever pneumonia (*%Pneumonia*) as related to relative GNI per capita. Relative GNI per capita is the ratio of a country’s GNI to the World Bank High GNI Index for the children’s birth years.

Fig 2 is a scatterplot of *%Pneumonia* in children three years old and older versus relative GNI for the studies listed in Table 4. The data for Fig 2 are shown in Table 6. Consistent with smaller pneumonia incidence in developed than developing countries [3,4], the trend is of *%Pneumonia* decreasing with increasing GNI per capita. However, Beijing, Tianjin and Shanghai, with relative GNIs at 0.98, 0.85 and 1.02 respectively, depart markedly from this trend as shown in Fig 2. These cities have *%Pneumonia* as high as or higher than countries with relative GNIs only half as great.

**Table 6:** *%Pneumonia* as related to relative GNI per capita, the ratio of a country’s GNI to the World Bank high income GNI for the children’s birth years.

Location	Relative GNI^a^	*%Pneumonia* (%)	Children’s birth years	Reference
**Texas, USA**	1.48	13.1	2002–2006	**Present Study**
Tucson AZ, USA	1.48	7.4	1980–1984	Castro-Rodriguez 1999 [25]
Germany^b^	1.13	13.5	1997–2005	Schnabel 2009 [23]
Germany^c^	1.11	6.9	1992–1996	Weigl 2003 [26]
Shanghai	1.02	33.2	2005–2008	Zhang 2013 [8]
**Beijing**	0.98	26.9	2005–2008	**Present Study**
**Tianjin**	0.85	29.1	2007–2010	**Present Study**
Spain	0.82	3.0	1995–1996	Garcés-Sánchez 2005 [28]
Hungary	0.57	24.6	1984–1989	Leonardi 2002 [22]
Slovak Republic	0.51	16.3		
Bulgaria	0.35	24.7		
Romania	0.34	27.9		
Chongqing	0.30	31.3	2005–2008	Zhang 2013 [8]

^a^ Relative GNI = GNI/High Income GNI.

^b^ Munich, Leipzig, Wesel, Bad Honnef.

^c^ Schleswig-Holstein.

Beijing has developed towards being a high GNI per capita city with unprecedented speed [64]. Although increasing GNI per capita is accompanied by changes that ameliorate risk factors for infectious diseases [65], it is possible that Beijing’s modernization and development has been so rapid that the positive effects have not yet had enough time to take root. Moreover, when a certain proportion of a population has been vaccinated, the reduced density of pathogens results in decreased incidence in the unvaccinated, or a “herd effect,” which further decreases the overall incidence [66].

### Limitations

This study is subject to the limitations of survey questionnaires, including recall bias and memory errors. In addition, reported “incidences” are an underestimate if medical attention was not sought for a sick child with pneumonia, but an overestimate if there was incentive, as there has been in China, for physicians to prescribe antibiotics [67]. The literature estimates of *VE* are for children ≤2 years old and not for older children; however, we have shown that for children aged 1 to 8, most pneumonia happens before age 3 (Fig 1 and S1 Fig). Literature *VE*s were derived from incidence rather than *%Pneumonia* data, and it is not known whether they can be applied to *%Pneumonia*. Our data do not include mortality statistics. However, we can estimate from the U5MR mortality rate of 0.25‰ in 2012 [1] that the pneumonia deaths in this sample of approximately 6,000 would have been 1.5, a mathematically negligible quantity. We also do not know the severity of pneumonia cases (whether the pneumonia required hospitalization or was invasive) or the number of cases per child. Finally, fluctuations in the severity of yearly influenza, a risk factor for pneumonia [68] and likely to increase pneumonia incidence especially in children 0–3 years-old, have been neglected.

## Conclusions

The proportion of Beijing children who have had pneumonia at least once by age 8, 26.9%, is similar to that of Tianjin. Because the Beijing *%Pneumonia* is mostly chest X-ray derived, it likely represents true pneumonia cases, but also may underestimate *%Pneumonia* for Beijing children. Beijing, Tianjin and Shanghai all have higher *%Pneumonia* than those of other comparably high GNI regions and countries. There is potential for reducing Beijing’s *%Pneumonia*. China, unlike other country locations with lower *%Pneumonia*, lacks both PCV and Hib national vaccination programs. Based on vaccine efficacies, it is estimated that national PCV and Hib vaccination would reduce Beijing’s *%Pneumonia* from 26.9% to 19.7% and 24.9% respectively.

However, Beijing would still have greater *%Pneumonia* than consistent with high GNI per capita. It is likely that Beijing’s severe air pollution [64], a known risk factor for pneumonia [69–71] must be addressed to achieve further reduction.

## Supporting information

S1 Fig Cumulative Lower Respiratory Tract Infection (LRTI) incidence as a function of age. (TIF)

S1 Table Ratio of pneumonia incidence in first 12, 24 and 36 months (M) of life to subsequent months. (DOCX)

S2 Table The proportion of children in each age group who have ever had pneumonia (*%Pneumonia*), Beijing, Tianjin and Texas. (DOCX)

S3 Table *VE*s of PCV and Hib by meta-analyses. S3A Table. Studies used in meta-analyses of PCV *VE*. S3B Table. Studies used in Theodoratou et al.’s meta-analysis of Hib *VE*.
(DOCX)

S1 Text Pneumonia diagnosis: clinical judgment versus chest X-ray confirmation. (DOCX)

## References

[1] Yan SJ, Zhu XN. Analysis of mortality rate and causes of death among children under 5 YO in Beijing from 2003 to 2012. Chinese J Prevent Med. 2014; 48(6):484–90. Chinese. 25219437

[2] Liu L, Johnson HL, Cousens S, Perin J, Scott S, Lawn JE, et al Global, regional, and national causes of child mortality: an updated systematic analysis for 2010 with time trends since 2000. Lancet. 2012; 379(9832):2151–61. 10.1016/S0140-6736(12)60560-1 22579125

[3] Madhi SA, De Wals P, Grijalva CG, Grimwood K, Grossman R, Ishiwada N, et al The burden of childhood pneumonia in the developed world: a review of the literature. Pediatr Infect Dis J. 2013; 32(3):e119–27. 23099423 10.1097/INF.0b013e3182784b26

[4] Rudan I, O'Brien KL, Nair H, Liu L, Theodoratou E, Qazi S, et al Epidemiology and etiology of childhood pneumonia in 2010: estimates of incidence, severe morbidity, mortality, underlying risk factors and causative pathogens for 192 countries. J Glob Health. 2013; 3(1):010401 23826505 10.7189/jogh.03.010401 PMC3700032

[5] Guan XJ, Silk BJ, Li WK, Fleischauer AT, Xing XS, Jiang XQ, et al Pneumonia incidence and mortality in mainland China: systematic review of Chinese and English literature, 1985–2008. PLoS ONE. 2010; 5:e11721 10.1371/journal.pone.0011721 20668535 PMC2909231

[6] Che DT, Zhou H, He JC, Wu B. Modeling the impact of the 7-valent pneumococcal conjugate vaccine in Chinese infants: an economic analysis of a compulsory vaccination. BMC Health Serv Res. 2014; 14:56 10.1186/1472-6963-14-56 24507480 PMC3918139

[7] Chen Y, Deng W, Wang SM, Mo QM, Jia H, Wang Q, et al Burden of pneumonia and meningitis caused by Streptococcus pneumoniae in China among children under 5 years of age: a systematic literature review. PLoS ONE. 2011; 6:e27333 10.1371/journal.pone.0027333 22110628 PMC3217934

[8] Zhang YP, Li BZ, Huang C, Yang X, Qian H, Deng QH, et al Ten cities cross-sectional questionnaire survey of children asthma and other allergies in China. Chinese Sci Bull. 2013; 58(34):4182–9.

[9] Nelson JC, Jackson M, Yu OC, Whitney CG, Bounds L, Bittner R, et al Impact of the introduction of pneumococcal conjugate vaccine on rates of community acquired pneumonia in children and adults. Vaccine. 2008; 26(38):4947–54. 10.1016/j.vaccine.2008.07.016 18662735

[10] World Bank. Indicator. [Cited 20 February 2016] [Internet]. 2016. http://data.worldbank.org/indicator/NY.GDP.MKTP.CDGNI. Scroll to “Economy & Growth, GDP per capita (current US$).”

[11] World Bank. GNI per capita, PPP (current international $). Scroll to “Economy & Growth, GNI per capita, PPP (current international $). [Cited 20 February 2016] [Internet]. 2016. http://data.worldbank.org/indicator/NY.GNP.PCAP.PP.CD

[12] Wikipedia. List of Chinese administrative divisions by GDP per capita. [Cited 20 February 2016] [Internet]. 2016. http://en.wikipedia.org/wiki/List_of_Chinese_administrative_divisions_by_GDP_per_capita

[13] Theodoratou E, Johnson S, Jhass A, Madhi SA, Clark A, Boschi-Pinto C, et al The effect of haemophilus influenzae type b and pneumococcal conjugate vaccines on childhood pneumonia incidence, severe morbidity and mortality. Int J Epidemiol. 2010; 39(suppl1):i172–85. 20348119 10.1093/ije/dyq033 PMC2845872

[14] O'Brien KL, Wolfson LJ, Watt JP, Henkle E, Deloria-Knoll M, McCall N, et al Burden of disease caused by Streptococcus pneumoniae in children younger than 5 years: global estimates. Lancet. 2009; 374(9693):893–902. 10.1016/S0140-6736(09)61204-6 19748398

[15] IVAC, International Vaccine Access Center, Johns Hopkins Bloomberg School of Public Health. Vaccine Information Management System (VIMS) Global Vaccine Introduction Report. May, 2016. [Cited 21 June 2016] [Internet]. 2016. http://www.jhsph.edu/research/centers-and-institutes/ivac/view-hub/IVAC_VIMS_Report%202016Mar_public_FINAL.pdf

[16] Richter SS, Diekema DJ, Heilmann KP, Dohrn CL, Riahi F, Doern GV. Changes in pneumococcal serotypes and antimicrobial resistance after introduction of the 13-valent conjugate vaccine in the United States. Antimicrob Agents Ch. 2014; 58:6484–9. 10.1128/AAC.03344-14 PMC4249410 25136018

[17] Qu F, Weschler LB, Sundell J, Zhang YP. Increasing prevalence of asthma and allergy in Beijing pre-school children: is exclusive breastfeeding for more than 6 months protective? Chinese Sci Bull. 2013; 58(34):4190–202.

[18] Wagner AL, Sun XD, Montgomery JP, Huang ZY, Boulton ML. The impact of residency and urbanicity on haemophilus influenzae type b and pneumococcal immunization in Shanghai children: a retrospective cohort study. PLoS ONE. 2014; 9:e97800 10.1371/journal.pone.0097800 24828814 PMC4020859

[19] Liu YN and Chinese Thoracic Society. Guidelines for diagnosis and treatment of community-acquired pneumonia. Chin J Tuberc Respir Dis. 2006; 29:651–5. Chinese.

[20] Lu C, Deng Q, Yu CWF, Sundell J, Ou C. Effects of ambient air pollution on the prevalence of pneumonia in children: Implication for national ambient air quality standards in China. Indoor Built Environ. 2014; 23(2):259–69.

[21] Liu W, Huang C, Hu Y, Zou ZJ, Zhao ZH, Sundell J. Association of building characteristics, residential heating and ventilation with asthmatic symptoms of preschool children in Shanghai: a cross-sectional study. Indoor Built Environ. 2014; 23(2):270–83.

[22] Leonardi GS, Houthuijs D, Nikiforov B, Volf J, Rudnai P, Zejda J, et al Respiratory symptoms, bronchitis and asthma in children of central and eastern Europe. Eur Respir J. 2002; 20(4):890–8. 12412680 10.1183/09031936.02.00260802

[23] Schnabel E, Sausenthaler S, Brockow I, Liese J, Herbarth O, Michael B, et al Burden of otitis media and pneumonia in children up to 6 years of age: results of the LISA birth cohort. Eur J Pediatr. 2009; 168(10):1251–7. 10.1007/s00431-008-0921-9 19159954

[24] Centers for Disease Control and Prevention. National, State, and Urban Area Vaccination Levels Among Children Aged 19–35 Months United States, 2001, 2002, 2003, 2004, 2005, 2006, 2007, 2008. MMWR Morb Mortal Wkly Rep. http://www.cdc.gov/vaccines/index.html.

[25] Castro-Rodriguez JA, Holberg CJ, Wright AL, Halonen M, Taussig LM, Morgan WJ, et al Association of radiologically ascertained pneumonia before age 3 yr with asthma-like symptoms and pulmonary function during childhood—a prospective study. Am J Respir Crit Care Med. 1999; 159(6):1891–7. 10.1164/ajrccm.159.6.9811035 10351936

[26] Weigl JA, Bader HM, Everding A, Schmitt HJ. Population-based burden of pneumonia before school entry in Schleswig-Holstein, Germany. Eur J Pediatr. 2003; 162(5):309–16. 12692711 10.1007/s00431-002-1140-4

[27] MacIntyre CR, McIntyre PB, Cagney M. Community-based estimates of incidence and risk factors for childhood pneumonia in western Sydney. Epidemiol Infect. 2003; 131(3):1091–6. 14959775 10.1017/s0950268803001365 PMC2870057

[28] Garcés-Sánchez M, Díez-Domingo J, Ballester SA, Peidró BC, García LM, Antón CV, et al Epidemiology of community-acquired pneumonia in children aged less than 5 years old in the autonomous community of Valencia (Spain). Proc Anales Pediatria (Barcelona, Spain: 2003). 2005; 63(2):125–30. 10.1157/13077454 16045871

[29] Li A, Sun Y, Liu Z, Xu X, Sun H, Sundell J. The influence of home environmental factors and life style on children’s respiratory health in Xi’an. Chinese Sci Bull. 2014; 59(17):2024–30.

[30] Harris M, Clark J, Coote N, Fletcher P, Harnden A, McKean M, et al British Thoracic Society guidelines for the management of community acquired pneumonia in children: update 2011. Thorax. 2011; 66(Suppl2):ii1–23. 21903691 10.1136/thoraxjnl-2011-200598

[31] Bradley JS, Byington CL, Shah SS, Alverson B, Carter ER, Harrison C, et al The management of community-acquired pneumonia in infants and children older than 3 months of age: clinical practice guidelines by the Pediatric Infectious Diseases Society and the Infectious Diseases Society of America. Clin Infect Dis. 2011; 53(7):e25–76. 10.1093/cid/cir531 21880587 PMC7107838

[32] Lynch T, Bialy L, Kellner JD, Osmond MH, Klassen TP, Durec T, et al A systematic review on the diagnosis of pediatric bacterial pneumonia: when gold is bronze. PLoS ONE. 2010; 5(8):e11989 10.1371/journal.pone.0011989 20700510 PMC2917358

[33] O'Grady K-AF, Torzillo PJ, Frawley K, Chang AB. The radiological diagnosis of pneumonia in children. Pneumonia: A Peer Reviewed Open Access Journal. 2014; 5:38–51. 10.15172/pneu.2014.5/482 PMC5922330 31641573

[34] Gupta D, Mishra S, Chaturvedi P. Fast breathing in the diagnosis of pneumonia—a reassessment. J Trop Pediatrics. 1996; 42(4):196–9. 10.1093/tropej/42.4.196 8816029

[35] Neuman MI, Scully KJ, Kim D, Shah S, Bachur RG. Physician assessment of the likelihood of pneumonia in a pediatric emergency department. Pediatr Emerg Care 2010; 26(11):817–22. 20944506 10.1097/PEC.0b013e3181fb0d95

[36] Grossman LK, Caplan SE. Clinical, laboratory, and radiological information in the diagnosis of pneumonia in children. Ann Emerg Med. 1988; 17(1):43–6. 3337414 10.1016/s0196-0644(88)80502-x

[37] Chiu SS, Ho PL, Khong PL, Ooi C, So LY, Wong WHS, et al Population-based incidence of community-acquired pneumonia hospitalization in Hong Kong children younger than 5 years before universal conjugate pneumococcal immunization. J Microbiol Immunol. 2016; 49(2):225–9. 10.1016/j.jmii.2014.05.007 25070281

[38] Rothrock SG, Green SM, Fanelli JM, Cruzen E, Costanzo KA, Pagane J. Do published guidelines predict pneumonia in children presenting to an urban ED? Pediatr Emerg Care. 2001; 17(4):240–3. ISSN: 0749-5161. 11493820 10.1097/00006565-200108000-00003

[39] Kuti BP, Adege SA, Oyelami OA. Can we predict which children with clinical pneumonia will have radiologic findings on chest radiograph? World J Med Med Sci. 2014; 2(3):1–12. ISSN: 2330-1341

[40] Roca A, Sigauque B, Quinto L, Morais L, Berenguera A, Corachan M, et al Estimating the vaccine-preventable burden of hospitalized pneumonia among young Mozambican children. Vaccine. 2010; 28(30):4851–7. 10.1016/j.vaccine.2010.03.060 20392430

[41] Benavides JA, Ovalle OO, Salvador GR, Gray S, Isaacman D, Rodgers GL. Population-based surveillance for invasive pneumococcal disease and pneumonia in infants and young children in Bogota, Colombia. Vaccine. 2012; 30(40):5886–92. 10.1016/j.vaccine.2012.03.054 22484295

[42] Njeze NR, Okwor C, Nzegwu M. A correlation between clinical and chest radiographic diagnosis of pneumonia in Nigerian children. Adv Biores. 2011; 2(2):18–21. ISSN: 0976-4585

[43] Andrade AL, Oliveira R, Vieira MA, Minamisava R, Pessoa V, Brandileone MC, et al Population-based surveillance for invasive pneumococcal disease and pneumonia in infants and young children in Goiania, Brazil. Vaccine. 2012; 30(10):1901–9. 10.1016/j.vaccine.2011.12.012 22178522

[44] Black SB, Shinefield HR, Ling S, Hansen J, Fireman B, Spring D, et al Effectiveness of heptavalent pneumococcal conjugate vaccine in children younger than five years of age for prevention of pneumonia. Pediatr Infect Dis J. 2002; 21(9):810–5. 12352800 10.1097/00006454-200209000-00005

[45] Lynch T, Platt R, Gouin S, Larson C, Patenaude Y. Can we predict which children with clinically suspected pneumonia will have the presence of focal infiltrates on chest radiographs? Pediatrics. 2004; 113(3):e186–9. 14993575 10.1542/peds.113.3.e186

[46] Smith KR, McCracken JP, Weber MW, Hubbard A, Jenny A, Thompson LM, et al Effect of reduction in household air pollution on childhood pneumonia in Guatemala (RESPIRE): a randomised controlled trial. Lancet. 2011; 378(9804):1717–26. 10.1016/S0140-6736(11)60921-5 22078686

[47] Ayalon I, Glatstein MM, Zaidenberg-Israeli G, Scolnik D, Tov AB, Sira LB, et al The role of physical examination in establishing the diagnosis of pneumonia. Pediatr Emerg Care. 2013; 29(8):893–6. 23903669 10.1097/PEC.0b013e31829e7d6a

[48] Bruce N, Weber M, Arana B, Diaz A, Jenny A, Thompson L, et al Pneumonia case-finding in the RESPIRE Guatemala indoor air pollution trial: standardizing methods for resource-poor settings. B World Health Organ. 2007; 85(7):535–44. 10.2471/BLT.06.035832 PMC2636369 17768502

[49] Turner C, Turner P, Carrara V, Burgoine K, Htoo STL, Watthanaworawit W, et al High rates of pneumonia in children under two years of age in a south east Asian refugee population. PLoS ONE. 2013; 8(1):e54026 10.1371/journal.pone.0054026 23320118 PMC3539989

[50] Arguedas A, Abdelnour A, Soley C, Jimenez E, Jimenez AL, Ramcharran D, et al Prospective epidemiologic surveillance of invasive pneumococcal disease and pneumonia in children in San Jose, Costa Rica. Vaccine. 2012; 30(13):2342–8. 10.1016/j.vaccine.2012.01.047 22300725

[51] Ho PL, Chiu SS, Chow FK, Mak GC, Lau YL. Pediatric hospitalization for pneumococcal diseases preventable by 7-valent pneumococcal conjugate vaccine in Hong Kong. Vaccine. 2007; 25(39–40):6837–41. 10.1016/j.vaccine.2007.07.039 17714837

[52] Clark JE, Hammal D, Spencer D, Hampton F. Children with pneumonia: how do they present and how are they managed? Arch Dis Child. 2007; 92(5):394–8. 10.1136/adc.2006.097402 17261579 PMC2083747

[53] Hazir T, Nisar YB, Qazi SA, Khan SF, Raza M, Zameer S, et al Chest radiography in children aged 2–59 months diagnosed with non-severe pneumonia as defined by World Health Organization: descriptive multicentre study in Pakistan. Brit Med J. 2006; 333(7569):629–31. 10.1136/bmj.38915.673322.80 16923771 PMC1570841

[54] Shah S, Bachur R, Kim D, Neuman MI. Lack of predictive value of tachypnea in the diagnosis of pneumonia in children. Pediatr Infect Dis J. 2010; 29(5):406–9. 20032805 10.1097/INF.0b013e3181cb45a7

[55] Ben Shimol S, Dagan R, Givon-Lavi N, Tal A, Aviram M, Bar-Ziv J, et al Evaluation of the World Health Organization criteria for chest radiographs for pneumonia diagnosis in children. Eur J Pediatr. 2012; 171(2):369–74. 10.1007/s00431-011-1543-1 21870077

[56] Pilishvili T, Chernyshova L, Bondarenko A, Lapiy F, Sychova I, Cohen A, et al Evaluation of the effectiveness of haemophilus influenzae type b conjugate vaccine introduction against radiologically-confirmed hospitalized pneumonia in young children in Ukraine. J Pediatr. 2013; 163(1-Suppl):12–8. 23773588 10.1016/j.jpeds.2013.03.025

[57] Sun YX, Sundell J. Early daycare attendance increases the risk for respiratory infections and asthma of children. J Asthma. 2011; 48(8):790–6. 10.3109/02770903.2011.604884 21838620

[58] Feikin DR, Scott JAG, Gessner BD. Use of vaccines as probes to define disease burden. Lancet. 2014; 383(9930):1762–70. 10.1016/S0140-6736(13)61682-7 24553294 PMC4682543

[59] Gessner BD, Feikin DR. Vaccine preventable disease incidence as a complement to vaccine efficacy for setting vaccine policy. Vaccine. 2014; 32(26):3133–8. 10.1016/j.vaccine.2014.04.019 24731817 PMC4772886

[60] Lucero MG, Williams G. Vaccine trial as "probe" to define the burden of pneumonia disease. Lancet. 2005; 365(9465): 1113–4. 10.1016/S0140-6736(05)71853-5 15794951

[61] Mulholland EK. Use of vaccine trials to estimate burden of disease. J Health Popul Nutr. 2004; 22(3): 257–67. ISSN1606-0997. 15609778

[62] Saadatian-Elahi M, Horstick O, Breiman RF, Gessner BD, Gubler DJ, Louis J, et al Beyond efficacy: The full public health impact of vaccines. Vaccine. 2016; 34(9): 1139–47. 10.1016/j.vaccine.2016.01.021 26808648 PMC11345718

[63] Lucero MG, Dulalia VE, Nillos LT, Williams G, Parreno RA, Nohynek H, et al Pneumococcal conjugate vaccines for preventing vaccine-type invasive pneumococcal disease and X-ray defined pneumonia in children less than two years of age. Cochrane DB Syst Rev. 2009; CD004977(3). 10.1002/14651858.CD004977.pub2 PMC6464899 19821336

[64] Zhang Y, Mo J, Weschler CJ. Reducing health risks from indoor exposures in rapidly developing urban China. Environ Health Perspect. 2013; 121(7):751–5. 10.1289/ehp.1205983 23665813 PMC3701998

[65] Feng XL, Theodoratou E, Liu L, Chan KY, Hipgrave D, Scherpbier R, et al Social, economic, political and health system and program determinants of child mortality reduction in China between 1990 and 2006: a systematic analysis. J Global Health. 2012; 2(1):010405. 10.7189/jogh.02.010405 PMC3484751 23198134

[66] Fine P, Eames K, Heymann DL. "Herd immunity": a rough guide. Clin Infect Dis. 2011; 52(7):911–6. 10.1093/cid/cir007 21427399

[67] Hvistendahl M. China takes aim at rampant antibiotic resistance. Science. 2012; 336(6083):795 10.1126/science.336.6083.795 22605727

[68] Nair H, Brooks WA, Katz M, Roca A, Berkley JA, Madhi SA, et al Global burden of respiratory infections due to seasonal influenza in young children: a systematic review and meta-analysis. Lancet. 2011; 378(9807):1917–30. 10.1016/S0140-6736(11)61051-9 22078723

[69] Burnett RT, Pope CA, Ezzati M, Olives C, Lim SS, Mehta S, et al An integrated risk function for estimating the global burden of disease attributable to ambient fine particulate matter exposure. Environ Health Perspect. 2014; 122(4):397–403. 10.1289/ehp.1307049 24518036 PMC3984213

[70] Pope CA. Respiratory disease associated with community air pollution and a steel mill, Utah Valley. Am J Public Health. 1989; 79(5):623–8. 2495741 10.2105/ajph.79.5.623 PMC1349506

[71] Dherani M, Pope D, Mascarenhas M, Smith KR, Weber M, Bruce N. Indoor air pollution from unprocessed solid fuel use and pneumonia risk in children aged under five years: a systematic review and meta-analysis. B World Health Organ. 2008; 86(5):390–8. 10.2471/BLT.07.044529 PMC2647443 18545742

