# Supplementary figures and images for: High pneumonia lifetime-ever incidence in Beijing children compared with locations in other countries, and implications for national PCV and Hib vaccination
Source: PLoS One. 2017 Feb 6;12(2):e0171438. doi: 10.1371/journal.pone.0171438 (PMC5293229; doi:10.1371/journal.pone.0171438)

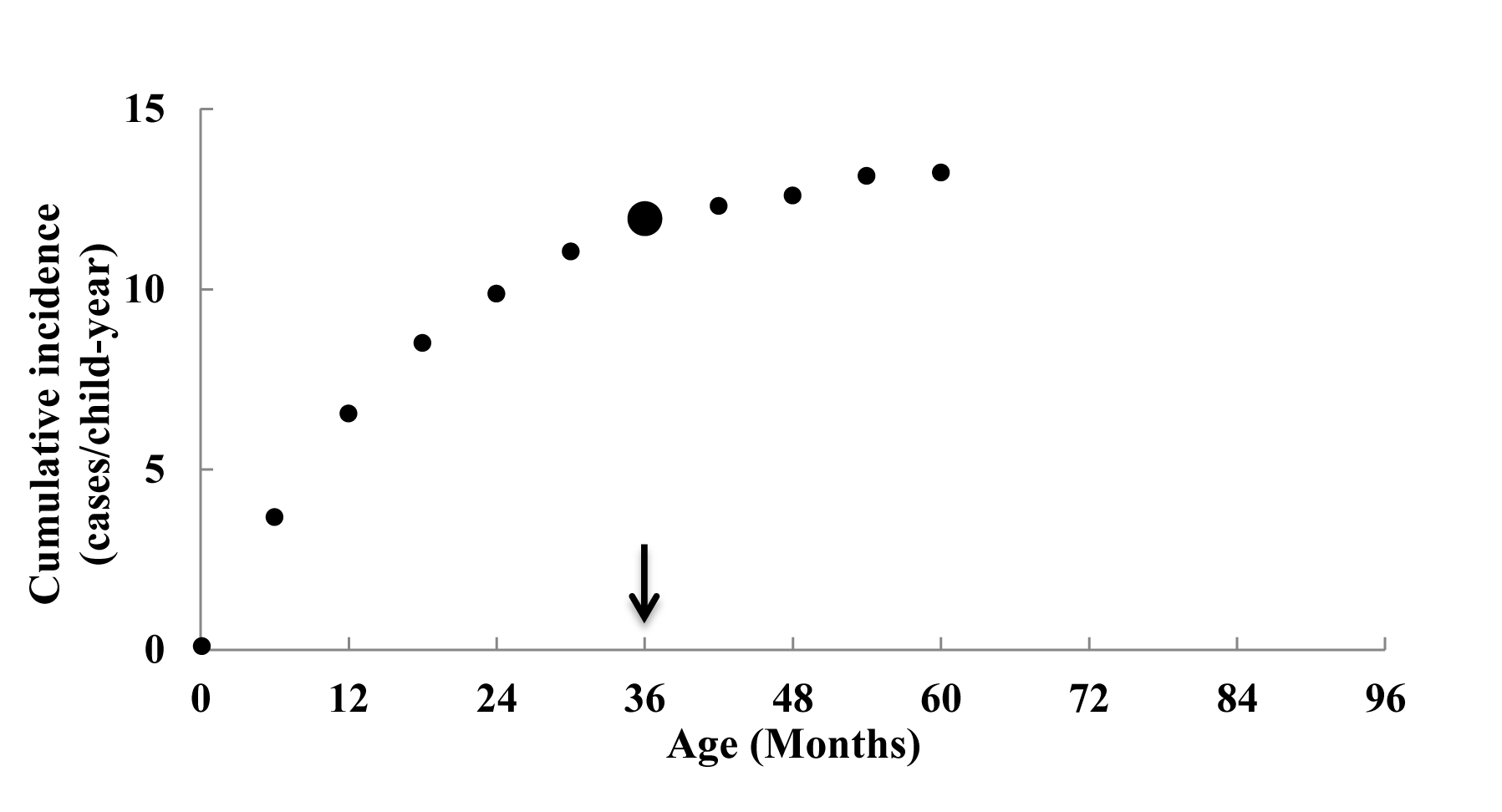

Supplement: S1 Fig — (TIF) [file pone.0171438.s001.tif]
