# Supplementary material for: High pneumonia lifetime-ever incidence in Beijing children compared with locations in other countries, and implications for national PCV and Hib vaccination
Source: PLoS One. 2017 Feb 6;12(2):e0171438. doi: 10.1371/journal.pone.0171438 (PMC5293229; doi:10.1371/journal.pone.0171438)
Supplement: S1 Table — (DOCX) [file pone.0171438.s002.docx]

**S1 Table.** **Ratio of pneumonia incidence in first 12, 24 and 36 months (M) of life to subsequent months**.

| **Region, Country** | **Years** | **Hospital (H)**  **Outpatient (O)**  **Both (B)** | **Early life incidence: cases/(1000child∙years)** | **Later life incidence cases/(1000child∙years)** | **Incidence ratio (Early/Later)** | **Comments** | **Reference** |
| --- | --- | --- | --- | --- | --- | --- | --- |
|  |  |  | **<36 M** | **36-59 M** |  |  |  |
| Vietnam | 2010 | H | 5.7 | 0.65 | 8.8 | Chest X-ray confirmed | Yoshida 2013 [1] |
| Bangladesh | 2004-2007 | B | 459 | 114 | 4.0 | Active surveillance,^a^  *N* = 12,000 | Arifeen 2009 [2] |
| North Carolina, USA | 1964-1975 | O | 140 | 45 | 3.1 | Passive surveillance, pediatricians’ offices^b^ | Denny 1986 [3] |
| Washington State, USA | 1998-2004 | H | 3.6 | 1.2 | 3.0 | Chest X-ray confirmed  Passive surveillance^b^ | Nelson 2008 [4] |
| Punjab, Pakistan | 2007-2008 | B | 343 | 130 | 2.6 | Active surveillance,^b^  *N* =5,570 | Owais 2010 [5] |
| Uruguay | 2007 | H | 15.5 | 6.0 | 2.6 | Chest X-ray consolidation | Hortal 2007 [6] |
| Canada | 1997-1998 | H | 8.9 | 3.6 | 2.5 | Retrospective data analysis | Petit 2003 [7] |
| Canada | 1989-1999 | B | 39.4 (6-35 M) | 25.3 | 1.6 | Retrospective data analysis | Petit 2003 [7] |
| Washington State, USA | 1998-2004 | B | 24.4 | 17.7 | 1.4 | Chest X-ray confirmed  Passive surveillance^b^ | Nelson 2008 [4] |
| Canada | 1989-1999 | B | 30.5 (6-35 M) | 21.8 | 1.4 | Retrospective data analysis | Petit 2003 [7] |
| Washington State, USA | 1998-2004 | O | 20.8 | 16.5 | 1.3 | Chest X-ray confirmed  Passive surveillance^b^ | Nelson 2008 [4] |
|  |  |  | **< 24 M** | **24 – 59 M** |  |  |  |
| Denmark | 1980-2001 | H | 1.22 | 0.25 | 4.9 | *Streptococcus pneumoniae* only | Mahon 2007 [8] |
| USA | 1996-1999 | H | 10.3 | 3.1 | 3.3 | Without PCV7 | Simonsen 2011 [9] |
| USA | 1997-1999 | H | 12.5 | 4.1 | 3.0 | Without PCV7 | Grijalva 2009 [10] |
| Latin America and Caribbean | 1992-2005 | B | 14.1 | 5.4 | 2.6 | Meta-analysis  (75% O, 25% H) | Gentile 2012 [11] |
| USA | 2005-2006 | H | 7.5 | 3 | 2.5 | With PCV7  national coverage | Simonsen 2011 [9] |
| Gambia | <1989 | B | 608 | 307 | 2.2 | Active surveillance, ^a^  *N* = 500 | Campbell 1989 [12] |
| USA | 2005-2006 | H | 8.6 | 4.4 | 2.0 | With PCV7  national coverage | Grijalva 2009 [10] |
|  |  |  | **< 12 M** | **12 – 59 M** |  |  |  |
| USA | 1997 | H | 11.7 | 3.8 | 3.1 | No PCV | Lee 2010 [13] |
|  | 2000 | H | 11.6 | 4.0 | 2.9 | No PCV |  |
|  | 2003 | H | 9.4 | 4.0 | 2.4 | PCV for <24M |  |
|  | 2006 | H | 9.1 | 3.9 | 2.3 | PCV for all |  |
|  | 1997-2006 | H | 10.4 | 3.9 | 2.7 | Average 1997-2006. Retrospective analysis of PCV efficacy |  |

^a^Active surveillance: Health worker visits family at regular time intervals (for example, every week or every two weeks). Thus, all children who may have pneumonia are identified.

^b^Passive surveillance: All hospital, clinic and MD records are reviewed, but pneumonia is diagnosed only in those children for whom parents sought medical attention. Active surveillance yields higher incidence than passive surveillance.

**References**

1. Yoshida LM, Nguyen HA, Watanabe K, Le MN, Nguyen AT, Vu HT, et al. Incidence of radiologically-confirmed pneumonia and haemophilus influenzae type b carriage before haemophilus influenzae type b conjugate vaccine introduction in central Vietnam. J Pediatr. 2013; 163(Suppl1):S38-43. [doi: 10.1016/j.jpeds.2013.03.029](http://dx.doi.org/10.1016/j.jpeds.2013.03.029) PMID: 23773592

2. Arifeen SE, Saha SK, Rahman S, Rahman KM, Rahman SM, Bari S, et al. Invasive pneumococcal disease among children in rural Bangladesh: results from a population-based surveillance. Clin Infect Dis. 2009; 48(Suppl2):S103-13. doi: 10.1086/596543 PMID: 19191605

3. Denny FW, Clyde WA. Acute lower respiratory tract infections in nonhospitalized children. J Pediatr. 1986; 108(5Pt1):635-46. doi: 10.1016/S0022-3476(86)81034-4 PMID: 3009769

4. Nelson JC, Jackson M, Yu OC, Whitney CG, Bounds L, Bittner R, et al. Impact of the introduction of pneumococcal conjugate vaccine on rates of community acquired pneumonia in children and adults. Vaccine. 2008; 26(38):4947-54. doi: 10.1016/j.vaccine.2008.07.016 PMID: 18662735

5. Owais A, Tikmani SS, Sultana S, Zaman U, Ahmed I, Allana S, et al. Incidence of pneumonia, bacteremia, and invasive pneumococcal disease in Pakistani children. Trop Med Int Health. 2010; 15(9):1029-36. doi: 10.1111/j.1365-3156.2010.02591.x PMID: 20636300

6. Hortal M, Estevan M, Iraola I, De Mucio B. A population-based assessment of the disease burden of consolidated pneumonia in hospitalized children under five years of age. Int J Infect Dis. 2007; 11(3):273-7. [doi: 10.1016/j.ijid.2006.05.006](http://dx.doi.org/10.1016/j.ijid.2006.05.006) PMID: 16997592

7. Petit G, De Wals P, Law B, Tam T, Erickson LJ, Guay M, et al. Epidemiological and economic burden of pneumococcal diseases in Canadian children. Can J Infect Dis. 2003; 14(4):215-20. PMC2094937 PMID: 18159460

8. Mahon BE, Ehrenstein V, Norgaard M, Pedersen L, Rothman KJ, Sorensen HT. Perinatal risk factors for hospitalization for pneumococcal disease in childhood: a population-based cohort study. Pediatrics. 2007; 119(4):e804-12. doi: 10.1542/peds.2006-2094 PMID: 17403823

9. Simonsen L, Taylor RJ, Young-Xu Y, Haber M, May L, Klugman KP. Impact of pneumococcal conjugate vaccination of infants on pneumonia and influenza hospitalization and mortality in all age groups in the United States. MBio. 2011; 2(1)e00309-10. doi: 10.1128/mBio.00309-10 PMID: 21264063

10. Grijalva CG, Griffin MR, Nuorti JP, Walter ND. Pneumonia hospitalizations among young children before and after introduction of pneumococcal conjugate vaccine - United States, 1997-2006. Morbid Mortal Weekly Rep. 2009; 58(1):1-4. ISSN: 0149-2195 PMID: 19145219

11. Gentile A, Bardach A, Ciapponi A, Garcia-Marti S, Aruj P, Glujovsky D, et al. Epidemiology of community-acquired pneumonia in children of Latin America and the Caribbean: a systematic review and meta-analysis. Int J Infect Dis. 2012; 16(1):e5-15. [doi: 10.1016/S0022-3476(86)81034-4](http://dx.doi.org/10.1016/S0022-3476(86)81034-4) PMID: 22056731

12. Campbell H, Byass P, Lamont AC, Forgie IM, O'Neill KP, Lloyd-Evans N, et al. Assessment of clinical criteria for identification of severe acute lower respiratory tract infections in children. Lancet. 1989; 1(8633):297-9. [doi: 10.1016/S0140-6736(89)91308-1](http://dx.doi.org/10.1016/S0140-6736(89)91308-1) PMID: 2563457

13. Lee GE, Lorch SA, Sheffler-Collins S, Kronman MP, Shah SS. National hospitalization trends for pediatric pneumonia and associated complications. Pediatrics. 2010; 126(2):204-13. doi: 10.1542/peds.2009-3109 PMID: 20643717
