# Supplementary material for: High pneumonia lifetime-ever incidence in Beijing children compared with locations in other countries, and implications for national PCV and Hib vaccination
Source: PLoS One. 2017 Feb 6;12(2):e0171438. doi: 10.1371/journal.pone.0171438 (PMC5293229; doi:10.1371/journal.pone.0171438)
Supplement: S2 Table — (DOCX) [file pone.0171438.s003.docx]

**S2 Table. The proportion of children in each age group who have ever had pneumonia (*%Pneumonia*), Beijing, Tianjin and Texas**.

| **Age** | **Beijing** | |  | **Tianjin** | |  | **Texas** | |
| --- | --- | --- | --- | --- | --- | --- | --- | --- |
| **(Years)** | ***N*** | ***%Pneumonia***  **(%)** |  | ***N*** | ***%Pneumonia***  **(%)** |  | ***N*** | ***%Pneumonia***  **(%)** |
| 1 | 24 | 20.8 |  | 56 | 23.2 |  | 202 | 8.4 |
| 2 | 120 | 14.2 |  | 98 | 27.6 |  | 339 | 11.2 |
| 3 | 1,336 | 25.4 |  | 528 | 28.6 |  | 384 | 11.2 |
| 4 | 1,598 | 27.2 |  | 1,104 | 31.8 |  | 380 | 10.8 |
| 5 | 1,476 | 27.4 |  | 1,363 | 27.6 |  | 353 | 15.3 |
| 6 | 921 | 27.6 |  | 1,621 | 28.7 |  | 407 | 15 |
| 7 | 75 | 28.0 |  | 1,466 | 29.9 |  | 387 | 11.9 |
| 8 | 34 | 32.4 |  | 517 | 24.4 |  | 331 | 14.8 |
| **3-8** | **5,440** | **26.9** |  | **6,599** | **28.9** |  | **2,242** | **13.1** |
