# Supplementary material for: High pneumonia lifetime-ever incidence in Beijing children compared with locations in other countries, and implications for national PCV and Hib vaccination
Source: PLoS One. 2017 Feb 6;12(2):e0171438. doi: 10.1371/journal.pone.0171438 (PMC5293229; doi:10.1371/journal.pone.0171438)
Supplement: S3 Table — S3A Table. Studies used in meta-analyses of PCV VE. S3B Table. Studies used in Theodoratou et al.’s meta-analysis of Hib VE. (DOCX) [file pone.0171438.s004.docx]

**S3 Table.** $\boldsymbol{VE}$**s of PCV and Hib by meta-analyses.**

**S3A Table. Studies used in meta-analyses of PCV** $\boldsymbol{VE}$**.**

| **Study** | **Used in these meta-analyses** | **Country** | **PCV valence** | ***N*_v_^a^** | ***n*_v_^b^** | ***I*_v_^c^** | ***N*_u_^d^** | ***n*_u_** | ***I*_u_** | $\boldsymbol{VE}$ **(%)**  **(95% CI)** |
| --- | --- | --- | --- | --- | --- | --- | --- | --- | --- | --- |
| Black 2002 [1] | O’Brien 2009 [2] | USA | PCV7 | 18,926 | - | - | 18,943 | - | - | 20.5 (4.4, 34.0) |
| Cutts 2005 [4] | Lucero 2009 [3]  O’Brien 2009 [2]  Theodoratou 2009 [5] | Gambia | PCV9 | 8,189 | 333 | 0.407 | 8,151 | 513 | 0.629 | 37 (27, 45) |
| Hansen 2006 [6] | O’Brien 2009 [2]  Lucero 2009 [3] | USA | PCV7 | - | - | - | - | - | - | 30.3 (10.7,45.7) |
| Klugman 2003 [7] | O’Brien 2009 [2]  Theodoratou 2009 [5] | South Africa | PCV9 | 19,922 | - | - | 19,914 | - | - | 20 (2,35) |
| Lucero 2009 [8] | O’Brien 2009 [2]  Lucero 2009 [3] | Philippines | PCV11 | 6,013 | 93 | 0.015 | 6,018 | 120 | 0.020 | 16 (-7.3,34.2) |
| Madhi 2005 [9] | Lucero 2009 [3] | South Africa | PCV9 | 18,633 | 251 | 0.013 | 18,626 | 303 | 0.016 | 17(2, 20) |

^a^*N*_v_ = Sample number vaccinated; subscript “v” is vaccinated.

^b^*n* = Number who have had pneumonia.

^c^*I* = Incidence = $n/N$.

^d^*N*_u_ = Sample number unvaccinated; subscript “u” is unvaccinated.

**S3B Table. Studies used in Theodoratou et al.’s meta-analysis of Hib** $\boldsymbol{VE}$**.**

| **Study** | **Country** | $\boldsymbol{VE}$ **(%)**  **(95% CI)** | **Type of Study** |
| --- | --- | --- | --- |
| de Andrade 2004 [10] | Brazil | 31 (-9, 57) | Case-control, 431 cases, 862 controls |
| Baqui 2007 [11] | Bangladesh | 32 (-2, 54) | Incident case-control |
| de la Hoz 2004 [12] | Colombia | 55 (7, 78) | Case-control, 385 cases, 770 controls |
| Gessner 2005 [13] | Indonesia | -12.0 (-248, 71) | RCT^a^, *N*=55,073 infants |
| Lagos 1996 [14] | Chile | Invasive only | **-** |
| Mulholland 1997 [15] | Gambia | 21.1 (4.6, 34.9) | RCT^a^, *N*=42,848 infants |

^a^ Randomized Controlled Trial.

**References**

1. Black SB, Shinefield HR, Ling S, Hansen J, Fireman B, Spring D, et al. Effectiveness of heptavalent pneumococcal conjugate vaccine in children younger than five years of age for prevention of pneumonia. Pediatr Infect Dis J. 2002; 21(9):810-5. doi: 10.1097/01.inf.0000027926.99356.4c PMID: 12352800
2. O'Brien KL, Wolfson LJ, Watt JP, Henkle E, Deloria-Knoll M, McCall N, et al. Burden of disease caused by Streptococcus pneumoniae in children younger than 5 years: global estimates. Lancet. 2009; 374(9693):893-902. doi: [10.1016/S0140-6736(09)61204-6](http://dx.doi.org/10.1016/S0140-6736(09)61204-6) PMID: 19748398
3. Lucero MG, Dulalia VE, Nillos LT, Williams G, Parreno RA, Nohynek H, et al. Pneumococcal conjugate vaccines for preventing vaccine-type invasive pneumococcal disease and X-ray defined pneumonia in children less than two years of age. Cochrane DB Syst Rev. 2009; CD004977(3). doi: 10.1002/14651858.CD004977.pub2 PMID: 19821336
4. Cutts FT, Zaman SM, Enwere G, Jaffar S, Levine OS, Oluwalana C, et al. Efficacy of nine-valent pneumococcal conjugate vaccine against pneumonia and invasive pneumococcal disease in The Gambia: randomised, double-blind, placebo-controlled trial. Lancet 2005; 365(9465): 1139-46. doi: [10.1016/S0140-6736(05)71876-6](https://dx.doi.org/10.1016/S0140-6736(05)71876-6) PMID: 15794968
5. Theodoratou E, Johnson S, Jhass A, Madhi SA, Clark A, Boschi-Pinto C, et al. The effect of haemophilus influenzae type b and pneumococcal conjugate vaccines on childhood pneumonia incidence, severe morbidity and mortality. Int J Epidemiol. 2010; 39(suppl1):i172-85. doi: 10.1093/ije/dyq033 PMID: 20348119
6. Hansen J, Black S, Shinefield H, Cherian T, Benson J, Fireman B, et al. Effectiveness of heptavalent pneumococcal conjugate vaccine in children younger than 5 years of age for prevention of pneumonia: updated analysis using World Health Organization standardized interpretation of chest radiographs. Pediatr Infect Dis J. 2006; 25(9):779-81. doi: 10.1097/01.inf.0000232706.35674.2f PMID: 16940833
7. Klugman KP, Madhi SA, Huebner RE, Kohberger R, Mbelle N, Pierce, Vaccine Trialists Group. A trial of a 9-valent pneumococcal conjugate vaccine in children with and those without HIV infection. N Engl J Med. 2003; 349(14): 1341-8. doi: 10.1056/NEJMoa035060 PMID: 14523142
8. Lucero MG, Nohynek H, Williams G, Tallo V, Simoes EA, Lupisan S, et al. Efficacy of an 11-valent pneumococcal conjugate vaccine against radiologically confirmed pneumonia among children less than 2 years of age in the Philippines: a randomized, double-blind, placebo-controlled trial. Pediatr Infect Dis J. 2009; 28(6): 455-62. doi: 10.1097/INF.0b013e31819637af PMID: 19483514
9. Madhi SA, Kuwanda L, Cutland C, Klugman KP. The impact of a 9-valent pneumococcal conjugate vaccine on the public health burden of pneumonia in HIV-infected and -uninfected children. Clin Infect Dis. 2005; 40(10): 1511-8. doi: 10.1086/429828 PMID: 15844075
10. de Andrade ALSS, de Andrade JG, Martelli CMT, Silva SAE, de Oliveira RM, Costa MSN, et al. Effectiveness of Haemophilus influenzae b conjugate vaccine on childhood pneumonia: a case-control study in Brazil. Int J Epidemiol. 2004; 33(1): 173-81. doi: 10.1093/ije/dyh025 PMID: 15075166
11. Baqui AH, El Arifeen S, Saha SK, Persson L, Zaman K, Gessner BD, et al. Effectiveness of Haemophilus influenzae type B conjugate vaccine on prevention of pneumonia and meningitis in Bangladeshi children: a case-control study. Pediatr Infect Dis. J 2007; 26(7): 565-71. doi: 10.1097/INF.0b013e31806166a0 PMID: 17596795
12. de la Hoz F, Higuera AB, Di Fabio JL, Luna M, Naranjo AG, de la Luz Valencia M, et al. Effectiveness of Haemophilus influenzae type b vaccination against bacterial pneumonia in Colombia. Vaccine. 2004; 23(1): 36-42. doi: 10.1016/j.vaccine.2004.05.017 PMID: 15519705
13. Gessner BD, Sutanti A, Lineham M, Djelantik IG, Fletcher T, Gerudug IK, et al. Incidences of vaccine-preventable Haemophilus influenzae type b pneumonia and meningitis in Indonesian children: hamlet-randomised vaccine-probe trial. Lancet. 2005; 365(9453): 43-52. doi: 10.1016/S0140-6736(04)17664-2 PMID: 15643700
14. Lagos R, Horwitz I, Toro J, San Martin O, Abrego P, Bustamante C, et al. Large scale, postlicensure, selective vaccination of Chilean infants with PRP-T conjugate vaccine: practicality and effectiveness in preventing invasive Haemophilus influenzae type b infections. Pediatr Infect Dis J. 1996; 15(3): 216-22. doi: 10.1097/00006454-199603000-00008 PMID: 8852909
15. Mulholland K, Hilton S, Adegbola R, Usen S, Oparaugo A, Omosigho C, et al. Randomised trial of Haemophilus influenzae type-b tetanus protein conjugate for prevention of pneumonia and meningitis in Gambian infants. Lancet. 1997; 349(9060): 1191-7. doi 10.1016/S0140-6736(96)09267-7 PMID: 9130939
