# Supplementary material for: High pneumonia lifetime-ever incidence in Beijing children compared with locations in other countries, and implications for national PCV and Hib vaccination
Source: PLoS One. 2017 Feb 6;12(2):e0171438. doi: 10.1371/journal.pone.0171438 (PMC5293229; doi:10.1371/journal.pone.0171438)
Supplement: S1 Text — (DOCX) [file pone.0171438.s005.docx]

**S1 Text. Pneumonia diagnosis: clinical judgment versus chest X-ray confirmation.**

**Clinical judgment: more sensitive, but subject to Type I errors**

Diagnosis by clinical judgment tends to include all true pneumonia (high sensitivity), but fails to exclude false cases of pneumonia (low specificity) [1].

No set of signs and symptoms has been shown to adequately predict a pneumonia-positive chest X-ray [2-6]. Some single clinical signs of respiratory distress are highly specific, but are seen in a too small fraction of cases to be adequately sensitive [1]. The lowest ratios of clinical to chest X-ray diagnoses are from well-trained, experienced MDs who use no specific algorithm or set of clinical criteria [7,8]. In resource-poor settings where poverty both decreases and delays health-care seeking, diagnostic sensitivity has priority over specificity, and consequently, the clinical to chest X-ray diagnosis ratio is larger [9,10]. In such settings, failing to diagnose and treat a true case of pneumonia (a Type II error) is viewed as a more consequential error than mistakenly treating a non-pneumonia as pneumonia (a Type I error) [11].

**Chest X-ray confirmation: specific, but subject to Type II errors**

Chest X-ray confirmation tends to include only true pneumonia cases (high specificity) but fails to include some true cases (low sensitivity) [12-14].

The particular chest X-ray findings specified for chest X-ray confirmation in China are either “alveolar consolidation” or “interstitial changes” [15,16]. Consolidation is precise [17], relatively easy to discern [18], and is the most reliable inter-observer finding [2,14,18-22]. It imparts strong probability to pneumonia diagnosis [2,23]. However, consolidation lacks sensitivity [21,23] for several reasons. Full consolidation requires time to develop in *S. pneumoniae* pneumonia [13]. Consolidation may at best be “patchy” particularly in viral pneumonia [24]. Moreover, it may not be present, even in cases of severe pneumonia [12,25] and even when apparent to one observer, not detected by another [21]. Finally, some pneumonias do not present radiographically with alveolar consolidation, but rather in ways detectable only by experienced radiologists [21,23,24]. “Interstitial change” is a less reliable sign than consolidation [24].

**Conclusion**

Whether by clinical criteria, chest X-ray confirmation or both, pneumonia diagnosis is impacted by health-care seeking behavior [26], especially on the timing of seeking health care [10].

Although agreement on chest X-ray is imperfect [22,23] even among experienced radiologists [9,27-29], chest X-ray remains the most widely used index test against which the diagnostic value of clinical signs is evaluated [5].

**References**

1. Rambaud-Althaus C, Althaus F, Genton B, D'Acremont V. Clinical features for diagnosis of pneumonia in children younger than 5 years: a systematic review and meta-analysis. Lancet Infect Dis. 2015; 15(4):439-50. doi: 10.1016/S1473-3099(15)70017-4 PMID: 25769269
2. Lynch T, Platt R, Gouin S, Larson C, Patenaude Y. Can we predict which children with clinically suspected pneumonia will have the presence of focal infiltrates on chest radiographs? Pediatrics. 2004; 113(3):e186-9. doi: 10.1542/peds.113.3.e186 PMID: 14993575
3. Mahabee-Gittens EM, Grupp-Phelan J, Brody AS, Donnelly LF, Bracey SE, Duma EM, et al. Identifying children with pneumonia in the emergency department. Clin Pediatr. 2005; 44(5):427-35. doi: 10.1177/000992280504400508 PMID: 15965550
4. Murphy CG, van de Pol AC, Harper MB, Bachur RG. Clinical predictors of occult pneumonia in the febrile child. Acad Emerg Med. 2007; 14(3):243-9. doi: 10.1197/j.aem.2006.08.022 PMID: 17242382
5. Lynch T, Bialy L, Kellner JD, Osmond MH, Klassen TP, Durec T, et al. A systematic review on the diagnosis of pediatric bacterial pneumonia: when gold is bronze. PLoS ONE. 2010; 5(8):e11989. doi: 10.1371/journal.pone.0011989 PMID: 20700510
6. Harris M, Clark J, Coote N, Fletcher P, Harnden A, McKean M, et al. British Thoracic Society guidelines for the management of community acquired pneumonia in children: update 2011. Thorax. 2011; 66(Suppl2):ii1-23. doi: 10.1136/thoraxjnl-2011-200598 PMID: 21903691
7. Grossman LK, Caplan SE. Clinical, laboratory, and radiological information in the diagnosis of pneumonia in children. Ann Emerg Med. 1988; 17(1):43-6. doi: 10.1016/S0196-0644(88)80502-X PMID: 3337414
8. Neuman MI, Scully KJ, Kim D, Shah S, Bachur RG. Physician assessment of the likelihood of pneumonia in a pediatric emergency department. Pediatr Emerg Care. 2010; 26(11):817-22. doi: 10.1097/PEC.0b013e3181fb0d95 PMID: 20944506
9. Bruce N, Weber M, Arana B, Diaz A, Jenny A, Thompson L, et al. Pneumonia case-finding in the RESPIRE Guatemala indoor air pollution trial: standardizing methods for resource-poor settings. B World Health Organ. 2007; 85(7):535-44. doi 10.1590/S0042-96862007000700011 PMID: 17768502
10. Nascimento-Carvalho CM, Madhi SA, OBrien KL. Review of guidelines for evidence-based management for childhood community-acquired pneumonia in under-5 years from developed and developing countries. Pediatr Infect Dis J. 2013; 32(11):1281-2. doi: 10.1097/INF.0b013e3182a4dcfa PMID: 24141800
11. Gove S, Pio A, Campbell H, Cattaneo A, Redd S, Rodman A, et al. WHO guidelines on detecting pneumonia in children. Lancet. 1991; 338(8780):1453-4. doi: [10.1016/0140-6736(91)92751-M](http://dx.doi.org/10.1016/0140-6736(91)92751-M) PMID: 1683434
12. Nizami SQ, Bhutta ZA, Hasan R, Husen YA. Role of chest X-ray in diagnosis of lower respiratory tract infections in children less than five years of age in community. Pak J Med Sci. 2005; 21(4):417-21. doi: 10.1197/j.aem.2006.08.022 ISSN:1682024X
13. Madhi SA, Klugman KP. World Health Organisation definition of "radiologically-confirmed pneumonia" may under-estimate the true public health value of conjugate pneumococcal vaccines. Vaccine. 2007; 25(13):2413-9. doi: 10.1016/j.vaccine.2006.09.010 PMID: 17005301
14. Williams GJ, Macaskill P, Kerr M, Fitzgerald DA, Isaacs D, Codarini M, et al. Variability and accuracy in interpretation of consolidation on chest radiography for diagnosing pneumonia in children under 5 years of age. Pediatr Pulmonol. 2013; 48(12):1195-200. doi: 10.1002/ppul.22806 PMID: 23997040
15. Liu YN and Chinese Thoracic Society. Guidelines for diagnosis and treatment of community-acquired pneumonia. Chin J Tuberc Respir Dis. 2006; 29:651-5. Chinese.
16. Guan XJ, Silk BJ, Li WK, Fleischauer AT, Xing XS, Jiang XQ, et al. Pneumonia incidence and mortality in mainland China: systematic review of Chinese and English literature, 1985-2008. PLoS ONE. 2010; 5:e11721. doi: 10.1371/journal.pone.0011721 PMID: 20668535.
17. Hansell DM, Bankier AA, MacMahon H, McLoud TC, Müller NL, Remy J. Fleischner society: glossary of terms for thoracic imaging. Radiology. 2008; 246(3):697-722. doi: 10.1148/radiol.2462070712 PMID: 18195376
18. Ben Shimol S, Dagan R, Givon-Lavi N, Tal A, Aviram M, Bar-Ziv J, et al. Evaluation of the World Health Organization criteria for chest radiographs for pneumonia diagnosis in children. Eur J Pediatr. 2012; 171(2):369-74. doi: 10.1007/s00431-011-1543-1 PMID: 21870077
19. Davies HD, Wang EE, Manson D, Babyn P, Shuckett B. Reliability of the chest radiograph in the diagnosis of lower respiratory infections in young children. Pediatr Infect Dis J. 1996; 15(7): 600-4. doi: 10.1007/s00431-011-1543-1 PMID: 8823854
20. Levinsky Y, Mimouni FB, Fisher D, Ehrlichman M. Chest radiography of acute paediatric lower respiratory infections: experience versus interobserver variation. Acta Paediatr. 2013; 102(7):e310-4. doi: 10.1111/apa.12249 PMID: 23565882
21. Test M, Shah SS, Monuteaux M, Ambroggio L, Lee EY, Markowitz RI, et al. Impact of clinical history on chest radiograph interpretation. J Hosp Med. 2013; 8(7):359-64. doi: 10.1002/jhm.1991 PMID: 23184766
22. Elemraid MA, Muller M, Spencer DA, Rushton SP, Gorton R, Thomas MF, et al. Accuracy of the interpretation of chest radiographs for the diagnosis of paediatric pneumonia. PLoS ONE. 2014; 9(8):e106051. doi: 10.1371/journal.pone.0106051 PMID: 25148361
23. O'Grady K-AF, Torzillo PJ, Frawley K, Chang AB. The radiological diagnosis of pneumonia in children. Pneumonia. 2014; 5:38-51. doi: [10.15172/pneu.2014.5/482](http://dx.doi.org/10.15172/pneu.2014.5/482)
24. Guo W, Wang J, Sheng M, Zhou M, Fang L. Radiological findings in 210 paediatric patients with viral pneumonia: a retrospective case study. Brit J Radiol. 2012; 85(1018):1385-9. doi: 10.1259/bjr/20276974 PMID: 22514104
25. Enwere G, Cheung YB, Zaman SMA, Akano A, Oluwalana C, Brown O, et al. Epidemiology and clinical features of pneumonia according to radiographic findings in Gambian children. Trop Med Int Health. 2007; 12(11):1377-85. doi: 10.1111/j.1365-3156.2007.01922.x PMID: 18045264
26. Rudan I, O'Brien KL, Nair H, Liu L, Theodoratou E, Qazi S, et al. Epidemiology and etiology of childhood pneumonia in 2010: estimates of incidence, severe morbidity, mortality, underlying risk factors and causative pathogens for 192 countries. J Glob Health. 2013; 3(1):010401. doi: 10.7189/jogh.03.010401 PMID: 23826505
27. Hansen J, Black S, Shinefield H, Cherian T, Benson J, Fireman B, et al. Effectiveness of heptavalent pneumococcal conjugate vaccine in children younger than 5 years of age for prevention of pneumonia: updated analysis using World Health Organization standardized interpretation of chest radiographs. Pediatr Infect Dis J. 2006; 25(9):779-81. doi: 10.1097/01.inf.0000232706.35674.2f PMID: 16940833
28. Johnson J, Kline JA. Intraobserver and interobserver agreement of the interpretation of pediatric chest radiographs. Emerg Radiol. 2010; 17(4):285-90. doi: 10.1007/s10140-009-0854-2 PMID: 20091078
29. Xavier-Souza G, Vilas-Boas AL, Fontoura M-SH, Araújo-Neto CA, Andrade SCS, Cardoso M-RA, et al. The inter-observer variation of chest radiograph reading in acute lower respiratory tract infection among children. Pediatr Pulmonol. 2013; 48(5):464-9. doi: 10.1002/ppul.22644 PMID: 22888091
